# Role of Regulatory T Cells (Treg) and the Treg Effector Molecule Fibrinogen-like Protein 2 in Alloimmunity and Autoimmunity

**DOI:** 10.5041/RMMJ.10209

**Published:** 2015-07-30

**Authors:** Andrzej Chruscinski, Hassan Sadozai, Vanessa Rojas-Luengas, Agata Bartczak, Ramzi Khattar, Nazia Selzner, Gary A. Levy

**Affiliations:** Multi-Organ Transplant Program, University Health Network, University of Toronto, Toronto, Canada

**Keywords:** Autoimmunity, FGL2, transplantation, Treg

## Abstract

CD4^+^CD25^+^Foxp3^+^ regulatory T cells (Treg) are critical to the maintenance of immune tolerance. Treg are known to utilize a number of molecular pathways to control immune responses and maintain immune homeostasis. Fibrinogen-like protein 2 (FGL2) has been identified by a number of investigators as an important immunosuppressive effector of Treg, which exerts its immunoregulatory activity by binding to inhibitory FcγRIIB receptors expressed on antigen-presenting cells including dendritic cells, endothelial cells, and B cells. More recently, it has been suggested that FGL2 accounts for the immunosuppressive activity of a highly suppressive subset of Treg that express T cell immunoreceptor with Ig and ITIM domains (TIGIT). Here we discuss the important role of Treg and FGL2 in preventing alloimmune and autoimmune disease. The FGL2–FcγRIIB pathway is also known to be utilized by viruses and tumor cells to evade immune surveillance. Moving forward, therapies based on modulation of the FGL2–FcγRIIB pathway hold promise for the treatment of a wide variety of conditions ranging from autoimmunity to cancer.

## HISTORICAL PERSPECTIVE ON REGULATORY T CELLS

A population of suppressive T cells was first postulated in the early 1970s by Gershon and Kondo, who discovered that some T cells could inhibit immune responses *in vitro*.^1^ These T cells were termed suppressor T cells and were found to be derived from a distinct population from helper T cells. The inability to define these cells more specifically led the scientific community to lose interest in the concept of “suppressor T cells.” In 1995, Sakaguchi identified CD25, the interleukin (IL)-2 receptor, as a marker for a population of T cells with the ability to inhibit autoimmune responses.^2^,^3^ Depletion of CD4^+^CD25^+^ T cells led to enhanced autoimmune and alloimmune responses, whereas adoptive transfer of these cells restored tolerance and prevented the development of autoimmune disease. The best characterized regulatory T cells are CD4^+^CD25^+^Foxp3^+^ T cells (Treg). Other regulatory T cell subsets are known to exist and include Tr1, Th3, CD8αβ^+^, CD8αα^+^, NKT cells, γδT cells, and double-negative T cells (DNT).^4^,^5^

In 2001, a mutation in the *Foxp3* gene, an X-linked transcription factor, was identified as the causative mutation in the Scurfy mouse, which displays a severe autoimmune phenotype.^6^,^7^ A mutation in *Foxp3* was then found to be responsible for the human disease, immunodysregulation polyendocrinopathy enteropathy X-linked syndrome (IPEX).^8^ This disorder is associated with autoimmune enteropathy, dermatitis, nail dystrophy, autoimmune endocrinopathies, and autoimmune skin conditions. This led to the discovery of *Foxp3* as the master regulator of Treg development and function. Once *Foxp3* is induced, its expression leads to expression of Treg signature genes including *Foxp3* itself.^9^,^10^ Investigators have focused their attention on defining the mechanism of action and ability of CD4^+^CD25^+^Foxp3^+^ Treg to induce tolerance. This review will focus on discussing the role of Treg in alloimmunity and autoimmunity, with an emphasis on the Treg effector molecule FGL2. In addition, we will discuss how therapies targeting the FGL2–FcγRIIB pathway may be used in various human diseases.

## MECHANISMS OF TREG-MEDIATED SUPPRESSION OF IMMUNE RESPONSES

**Figure 1:**
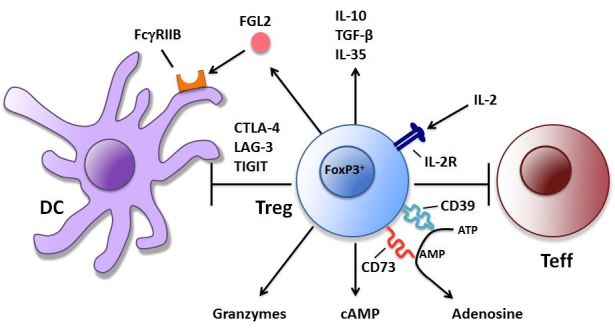
Mechanisms of Treg-mediated Immune Suppression Treg suppress immune responses through molecular pathways that act directly on T cells or indirectly through antigen-presenting cells such as dendritic cells. These molecular mechanisms are described in Table 1. FGL2 binds to FcγRIIB on dendritic cells to inhibit dendritic cell maturation. DC, dendritic cell; IL-2R, IL-2 receptor; Teff, effector T cell; Treg, regulatory T cell.

The CD4^+^CD25^+^Foxp3^+^ Treg have been shown to employ multiple mechanisms to inhibit immune responses.^11^,^12^ Some of these mechanisms directly inhibit T effector cells, while others indirectly inhibit T effector cells by acting on antigen-presenting cells (APC) such as dendritic cells (DC) (Figure 1, Table 1). Molecules credited with contributing to Treg suppressive activity include IL-10, TGF-β, CD39/CD73, IL-35, and FGL2.^12^,^13^ Treg have been shown to bind IL-2 through the high-affinity IL-2 receptor, thereby depriving dividing T cells of IL-2 and promoting apoptosis in these cells.^14^ Additional studies have shown that Treg can release granzyme B that can result in the apoptosis of activated T cells.^15^ Certain Treg subsets secrete adenosine that can act through A2A receptors to promote anergy in T cells.^16^ Treg have also been shown to produce high local levels of cAMP that can be transferred to T cells via gap junctions.^17^

**Table 1:** Treg Effector Molecules.

Effector	Cell Type	Ligand/Receptor	Target Cell	Mechanism
CTLA-4	Treg	B7 molecules (CD80/CD86)	DC	Inhibition of DC activation through the trans-endocytosis and degradation of CD80 and CD86 molecules by Treg Sterically hinders the association of naïve T cells with DC through co-ordinated activity with LFA-1 Negative regulation of effector T cell survival by signaling through Foxp3
IL-2	Activated T cells	High-affinity IL-2 receptor	Treg	IL-2 deprivation by Treg in low-affinity TCR and antigen–MHC interactions induce T cell apoptosis
TIGIT	Treg, T cells, NK cells	CD155 (PVR), CD112 (PVRL2)	DC	Inhibition of IL-12 (p40) production by DCBinds CD155 (PVR) and CD112 (PVRL2) on APCs Increases IL-10 expression inducing tolerogenic DC which suppress T cell proliferation and IFN-γ production
LAG-3	Treg	MHC-II	DC	Inhibits DC maturation Inhibits co-stimulation of naïve T cells by DC
CD39/CD73	Activated Treg	Treg	Activated T cells, DC	CD39 converts ATP in the extracellular space into ADP and AMP, decreasing inflammation CD39 increases suppressive activity of Treg CD73 converts AMP to adenosine which inhibits DC function and activated T cells
IL-10	Treg	IL-10R	T cells, DC	Inhibits T cell proliferation, decreases production of IL-2, TNF-α, and IL-5 Impairs Th1 responses by inhibiting DC activation and inhibiting secretion of IL-2
TGF-β	Treg	TGF-βR	T cells	Direct suppression of effector T cells Inhibits cytokine production and cytotoxic function of T cells
IL-35	Treg	IL-35R	Naïve T cells, DC	Direct inhibition of T cell proliferation Induction of naïve T cells to become activated IL-35 Treg
Gzmb	Treg	Perforin-independent entry into target cell	Activated T cells, DC	Induction of apoptosis in target cells
FGL2	T cells, Treg, activated Treg	FcγRIIB/RIII	DC	Inhibition of DC maturation Suppression of Th1 and Th17 effector T cell responses

ADP, adenosine diphosphate; AMP, adenosine monophosphate; APC, antigen-presenting cell; ATP, adenosine triphosphate; CTLA-4, cytotoxic T lymphocyte-associated protein 4; DC, dendritic cell; FGL2, fibrinogen-like protein 2; Foxp3, forkhead box p3; Gzmb, granzyme B; IL, interleukin; LAG-3, lymphocyte activation gene 3; LFA-1, lymphocyte function-associated antigen 1; MHC, major histocompatibility complex; PVR, poliovirus receptor; PVRL, poliovirus receptor ligand; TCR, T cell receptor; TGF, transforming growth factor; TIGIT, T cell immunoreceptor with Ig and ITIM domains.

CD4^+^CD25^+^Foxp3^+^ Treg can also modulate the activity of DC though the expression of various cell surface molecules. Treg are known to express high levels of cytotoxic T lymphocyte-associated protein 4 (CTLA-4), which can downregulate the expression of the important co-stimulatory molecules CD80 and CD86 on DC. CTLA-4 is thought to bind directly to and capture these co-stimulatory molecules in a process known as trans-endocytosis.^18^ The LAG-3 molecule is a surface molecule of Treg that is thought to inhibit DC by binding to major histocompatibility complex (MHC) class II. This binding transmits an inhibitory signal that prevents DC maturation and reduces co-stimulatory activity.^19^ More recently, the surface molecule T cell immunoreceptor with Ig and ITIM domains (TIGIT) has been identified as a marker of a highly suppressive population of CD4^+^CD25^+^Foxp3^+^ Treg. The receptor for TIGIT is the poliovirus receptor (PVR) on DC.^20^ A recent report by Joller et al. demonstrated that TIGIT^+^ Treg express large amounts of the Treg effector molecule FGL2. Furthermore, the suppression of Th1 and Th17 but not Th2 responses by TIGIT^+^ Treg was dependent on FGL2.^21^ *In vivo*, FGL2 was critical in the control of effector T cell expansion by TIGIT^+^ Treg in lymphopenic hosts and in controlling a number of inflammatory diseases including colitis.^21^

## FIBRINOGEN-LIKE PROTEIN 2 IS A TREG EFFECTOR MOLECULE

Fibrinogen-like protein 2 (FGL2), or fibroleukin, was originally cloned from a cDNA library made from cytotoxic T cells. The *fgl2* gene is localized to the proximal region of chromosome 5 in mice, 7q11.23 in humans, and 9 in pigs.^22^,^23^ The longest open reading frame encodes a 432-, 439-, and 442-amino acid protein in mice, humans, and pigs, respectively.^23^ The FGL2 protein is highly homologous between species (78% homology between mouse and human and 89% homology between pig and human), with greater conservation at the carboxyl terminus. The fully glycosylated FGL2 protein shows molecular sizes of approximately 65–70 kilo-Daltons (kDa) and 260–280 kDa under reducing and non-reducing conditions, respectively, suggesting that it has a tetrameric structure.^24^–^26^ Amino acid sequence analysis of FGL2 reveals an N-terminal hydrophobic motif, predicted as either the transmembrane domain or a signal peptide, with a carboxyl-terminal domain highly homologous (36%) to the fibrinogen beta and gamma subunits, the so-called fibrinogen-related domain (FRED).^23^,^25^,^27^ Thus FGL2 is classified as a member of the fibrinogen-related family of proteins, which also includes tenascin, angiopoietin, and ficolin.^28^

Fibrinogen-like protein 2 has been shown to exist both as a membrane-bound protein and as a secreted molecule. The biological function of FGL2 was first documented in a murine fulminant hepatitis model, in which FGL2 expression is induced in macrophages and endothelial cells, leading to a novel tissue factor-independent prothrombin cleaving activity.^27^ This prothrombinase activity is associated with a membrane form of FGL2, which is detectable by cell surface immunofluorescence staining.^29^ Serine 89 of FGL2 is critical for the prothrombinase activity, which also requires calcium, phospholipids, and factor Va for its full activity.^30^ The prothrombinase activity of FGL2 has been implicated in the pathogenesis of viral heaptitis, fetal loss, and rejection in xenografts.^23^,^31^,^32^

In addition to their role in coagulation, fibrinogen and fibrinogen-related proteins including FGL2 have been shown to have a role in control of immune responses.^33^–^35^ For example, binding of fibrinogen to its receptor MAC-1 expressed on macrophages leads to macrophage activation, and ligation to TLR4 leads to expression of MCP1.^36^ The secreted form of FGL2 is known to be produced by CD4^+^ and CD8^+^ T cells^25^ and is highly expressed by CD4^+^CD25^+^Foxp3^+^ Treg.^10^,^13^,^37^ In addition to CD4^+^ Treg, an immunosuppressive subset of CD8αα^+^ intraepithelial lymphocytes found in the small intestine was found to express *fgl2* mRNA.^38^ Furthermore, Li et al. recently demonstrated in a rat cardiac transplant model that tolerogenic CD8^+^CD45RC^low^ Treg expressed high levels of *fgl2* mRNA compared to naïve CD8^+^ Treg.^39^ A list of these FGL2-expressing Treg and their properties is shown in Table 2.

**Table 2:** FGL2-expressing Regulatory T Cells.

Molecule	CD8αα	CD8^+^CD45RC^low^	DNT cells	CD4^+^Foxp3^+^
TCR	αβ, MHC-I/II restricted	αβ, MHC-I restricted	αβ, MHC-II restricted	αβ, MHC-II-restricted
Co-receptor	CD8αα Small subset express CD4 or CD8	CD8αβ	Absent	CD4
Origin	Lin-cells in intestinal epithelium, cryptopatches	Unknown	Thymus, periphery	Thymus
Development	Thymus (induced IEL from conventional T cells) Thymus-independent	CD40-Ig treatment	Thymus-dependent (DC^+^, IL-12^+^, IL-15), thymus-independent	Thymus (tTreg) Induced in the periphery (pTreg)
Specificity	Self-antigen, foreign antigen, oligoclonal		Polyclonal	
Markers	CD69, FasL, granzymes, CD122, B220, NK-Like receptors Negative for CD2, CD5, CD28, LFA-1, mostly Thy1-negative	CD45RC^low^, Foxp3, GITR, IL-10, and IL-13	CD25, CD28, FasL, perforin, CTLA-4 Negative for NK1.1, Foxp3	CD25^high^, GITR, CTLA-4, OX-40, TIGIT, CD39/CD73, IL-35, PD-1, Gzmb
Cytokine expression	Low CD5 in intestine, TGF-β3, LAG-3, FGL2	IFN-γ, IDO, FGL2	FGL2, IFN-γ (not IL-2)	IL-10, TGF-β, FGL2
Target cell/specialization	Homeostasis in intestine (food and microbes in lamina propria) More common in the small intestine	Interaction with plasmacytoid DC to suppress CD4^+^ T cell activity Accumulated in the graft and spleen	LPS-activated DC CD8 and CD4 T cells Mature and immature DC B cells	DC T cells
Mechanisms	Inhibitory NK receptors CD8αα recruitment of LAT and LCK from the TCR	Contact-dependent- FGL2-mediated suppression of T cell proliferation Contact-independent IDO-mediated suppression	Trogocytosis and CD8^+^ T cell (FasL) mediated killing CTLA-4 dependent downregulation of DC activation DC apoptosis through Fas:FasL	DC inhibition by sequestration of CD80/CD86 T cell deprivation of IL-2 Inhibition of DC maturation Adenosine inhibition, impeding T cell effector and DC activity Anti-inflammatory Induction of apoptosis in target cells

CTLA-4, cytotoxic T lymphocyte-associated protein 4; DC, dendritic cell; DNT, double negative T cell; FasL, fas ligand; FGL2, fibrinogen-like protein 2; Foxp3, forkhead box p3; GITR, glucocorticoid-induced TNFR family-related gene; Gzmb, granzyme B; IDO, indoleamine 2,3-deoxygenase; IEL, intraepithelial lymphocytes; IFN-γ, interferon gamma; Ig, immunoglobulin; IL, interleukin; LAG-3, lymphocyte activation gene 3; LAT, linker for activation of T cells; LCK, lymphocyte-specific protein tyrosine kinase; LFA-1, lymphocyte function-associated antigen 1; Lin, lineage; LPS, lipopolysaccharide; MHC, major histocompatibility complex; NK, natural killer; PD-1, programmed cell death-1; TCR, T cell receptor; TGF-β3, transforming growth factor beta 3; Thy1, thymocyte antigen; TIGIT, T cell immunoreceptor with Ig and ITIM domains.

**Figure 2:**
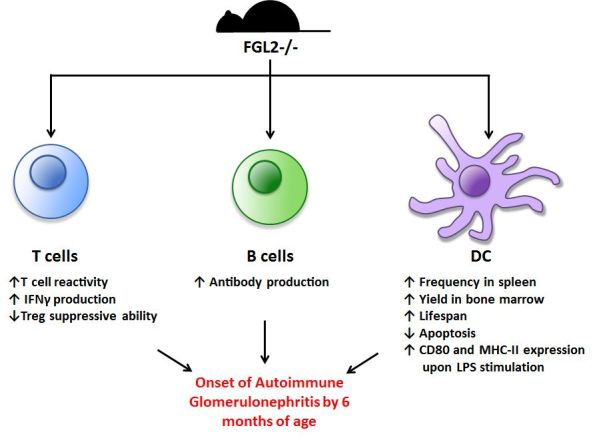
Immunoregulatory Function of FGL2 Mice deficient in FGL2 (*fgl2*^−/−^) have enhanced T cell, B cell, and DC function as shown in the figure. The *fgl2*^−/−^ mice develop autoimmune glomerulonephritis as they age reflective of chronic immune activation. DC, dendritic cell; LPS, lipopolysaccharide.

In an early report, we demonstrated that FGL2 directly inhibits T cell proliferation in response to various stimuli (alloantigen, ConA, and anti-CD3/anti-CD28 mAbs) and promotes a Th2 response. Furthermore, FGL2 was found to inhibit the maturation of bone marrow-derived DC (BMDC), reducing their ability to stimulate T cells in mixed lymphocyte reaction (MLR) co-cultures.^40^ In order to elucidate further the role of FGL2, we generated mice with a targeted deletion of *fgl2* (*fgl2*^−/−^) and found that these mice have increased T cell, B cell, and DC reactivity compared to *fgl2*^+/+^ wild-type mice (Figure 2).^13^ Furthermore, Treg isolated from *fgl2*^−/−^ mice had impaired ability to suppress effector T cell proliferation. The *fgl2*^−/−^ mice also develop autoimmune glomerulonephritis as they age, likely related to the state of immune activation.

The mechanisms through which FGL2 exerts its immunomodulatory function have been an area of active research. We and others have shown that FGL2 binds to FcγRIIB and RIII.^41^ FcγRIIB is a low-affinity inhibitory receptor with an immunoreceptor tyrosine-based inhibition motif (ITIM), which is widely expressed on myeloid cells, DC, and B cells.^42^,^43^ It recruits phosphatases, such as SHIP (Src homology domain 2–containing inositol phosphatase) to inhibit immunoreceptor tyrosine-based activation motif (ITAM) signaling. Self-ligation and cross-linking of FcγRIIB also results in B cell apoptosis, and B cell-specific FcγRIIB knockout mice have increased antibody responses with an enhanced susceptibility to arthritis.^43^ Interestingly, FcγRIIB^−/−^ mice develop autoimmune glomerulonephritis similar to *fgl2*^−/−^ mice.^44^,^45^ We have reported that binding of FGL2 to FcγRIIB on B cells leads to B cell apoptosis and that A20IIA1.6 cells, which lack FcγRIIB, are protected from FGL2-induced apoptosis.^41^ Similarly, FGL2 is ineffective at inhibiting bone marrow-derived DC maturation in FcγRIIB^−/−^ mice, further supporting the concept that the FGL2–FcγRIIB interaction is the major pathway accounting for the immunosuppressive activity of FGL2.^41^

## ROLE OF TREG AND FGL2 IN TRANSPLANTATION/ALLOIMMUNITY

CD4^+^CD25^+^Foxp3^+^ Treg are known to play a critical role in the induction and maintenance of tolerance in solid organ transplantation. In experimental animal models, we and others have shown that depletion of Treg prevents the development of tolerance.^39^,^46^–^48^ In order to investigate the role of Treg in tolerance, we established a mouse model of rapamycin-induced allograft tolerance. In this model, a short course of rapamycin (10 doses of 0.4 mg/kg over 16 days) led to long-lasting tolerance of heart allografts (>100 days). Tolerant mice were found to have an expansion of splenic and intragraft Foxp3^+^FGL2^+^ Treg compared with rejecting mice. Importantly, depletion of Treg with an anti-CD25 antibody (PC61) during rapamycin induction abrogated allograft tolerance and led to rejection of allografts. 49

In preclinical rodent models, treatment with donor-specific Treg has been shown to prolong allograft survival and induce tolerance.^50^ For these studies, donor-specific Treg were generated that were specific for direct antigen recognition. Regulatory T cells specific for both direct and indirect antigen presentation may have additional benefit in preventing chronic as well as acute rejection.^51^ These studies have stimulated interest in bringing Treg-based therapies to the clinic for use in clinical transplantation.^50^

In human solid organ transplantation, numerous studies have identified an association between Treg and tolerance.^52^ A role for rapamycin in promoting Treg has also been observed in liver transplant recipients who were switched from a calcineurin inhibitor to rapamycin. In this study, rapamycin treatment led to significant increases in peripheral blood mononuclear cells (PBMC) Treg levels and to increases in the intragraft Foxp3-to-CD3 ratio.^53^

**Figure 3:**
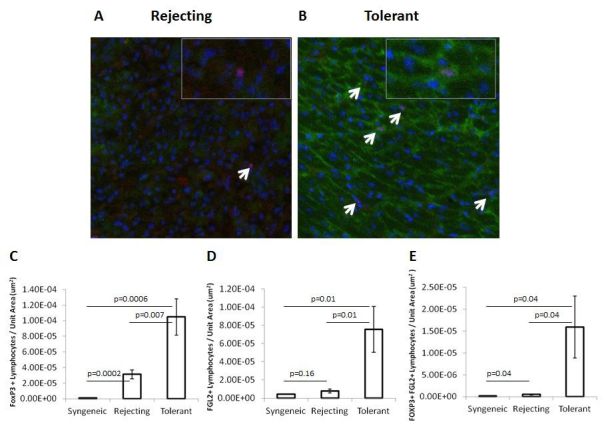
Co-expression of FGL2 and Foxp3 in Treg in Tolerant Allografts **Panel A and B**: Transplanted hearts were harvested from (**A**) rejecting mice or from (**B**) tolerant C3H mice at POD 100 and subsequently immunostained for Foxp3 (red) and FGL2 (green) (magnification 200×). Nuclei were visualized with DAPI (blue). Tolerant mice had significantly increased numbers of Foxp3^+^ Treg (white arrow). Whereas Foxp3^+^ Treg from tolerant mice largely expressed FGL2, Foxp3^+^, Treg in rejecting mice did not express FGL2. Inset shows a FGL2^−^ Treg in a rejecting allograft and a FGL2^+^ Treg in a tolerant allograft (magnification 1000×). **Panel C–E:** Morphometric analysis of the immunostained sections was performed using a Definiens analysis assessing the (**C**) number of Foxp3^+^/μm^2^,**(D)** FGL2^+^/μm^2^, and **(E)** Foxp3^+^FGL2^+^/μm^2^. Cardiac myocytes were excluded from analysis using size exclusion. Lymphocytes were defined based on size of 10 microns or less. The morphometric analysis of heart allografts is from 6 rejecting mice, 7 tolerant mice, and 3 syngeneic mice, with 4 serial sections taken at multiple levels of the heart. Data are expressed as the mean±SEM. Statistical significance was assessed using Student’s *t* test. Copyright (2014) John Wiley and Sons. Used with permission from Urbanellis P, Shyu W, Khattar R, et al.^49^

As a pivotal Treg effector molecule, FGL2 has been shown to be necessary for tolerance induction. We observed that an antibody to FGL2 enhanced proliferation in mixed lymphocyte reactions *in vitro*, consistent with the known immunomodulatory activity of FGL2.^49^ When an anti-FGL2 antibody was given concurrently with rapamycin in our mouse transplant model, it blocked tolerance induction. Unlike anti-CD25 (PC61), the anti-FGL2 antibody did not deplete intragraft Treg, consistent with FGL2 acting as a secreted molecule. In order to verify that FGL2-expressing Treg were associated with transplant tolerance, we performed dual-labeling studies in syngeneic, rejecting, and tolerant mouse heart grafts to identify Foxp3^+^ and FGL2^+^ cells (Figure 3).^49^ Staining for *Foxp3* was mainly observed in the nuclear compartment and co-localized with DAPI, whereas FGL2 staining was mainly observed in the membrane and cytoplasmic compartments. Compared with both syngeneic and rejecting allografts, tolerant allografts were associated with higher numbers of Foxp3^+^ cells and FGL2^+^ cells. Of interest, dual staining Foxp3^+^/FGL2^+^ cells, indicative of FGL2-expressing Treg, were almost exclusively found in the tolerant heart allografts. These results support our contention that FGL2^+^ Treg may be the critical cells that are important for maintenance of transplant tolerance. The FGL2 molecule has also been shown to be a critical Treg effector in a rat model of transplant tolerance induced by co-stimulation blockade. In this model, tolerance was dependent on CD8^+^ Treg, and FGL2 was necessary for contact-dependent inhibition of effector T cells by CD8^+^ Treg.^39^

We have now developed recombinant FGL2 (rFGL2) as a potential therapeutic in transplantation. Studies in a mouse skin transplant model have revealed that rFGL2 can prolong skin graft survival.^41^ Grafts remained viable as long as rFGL2 was administered. However, following cessation of therapy, grafts were rejected within 3–9 days, with histological evidence of cell-mediated rejection. We have also observed the same finding when mouse recipients of cardiac allografts were treated with rFGL2: as long as rFGL2 was administered the grafts survived, but soon after treatment stopped the grafts were rejected. It is currently unclear as to why rFGL2 does not promote tolerance by itself, and we are currently evaluating alternative delivery modalities and dosing schedules that would enhance the tolerogenic potential of rFGL2 in preclinical models.

In order to determine if continuous expression of FGL2 can promote transplant tolerance, we developed FGL2-overexpressing (*fgl2*^Tg^) mice. In these mice, FGL2 is expressed ubiquitously, and the mice have plasma levels of FGL2 that are 6–7-fold higher than wild-type mice. CD4^+^CD25^+^Foxp3^+^ Treg from *fgl2*^Tg^ mice have enhanced suppressive activity compared with Treg from littermate controls in a standard Treg suppression assay. Interestingly, 50% of these *fgl2*^Tg^ mice accept fully MHC-mismatched cardiac allografts without the need for immunosuppression, and tolerant allografts are associated with increased numbers of intragraft Treg (unpublished data).

Long-lasting tolerance has also been established in a rodent transplant model with FGL2 overexpression using a viral vector. In this model, an adenovirus-associated virus was used to overexpress FGL2 (AAV-FGL2) in recipients 30 days prior to transplantation, and three of eight recipients that received the AAV-FGL2 developed tolerance to heart allografts. The CD45RA^+^ cells from the tolerant recipients could transfer tolerance to sub-lethally irradiated recipients suggesting that generation of regulatory B cells could be involved in transplant tolerance mediated by FGL2 overexpression.^54^

## ROLE OF TREG AND FGL2 IN AUTOIMMUNITY

Studies have demonstrated that immune dysregulation plays an important role in both the initiation and progression of autoimmune disease (AID).^55^ Furthermore, it has been shown that reduced frequency and function of Treg are associated with the development of AID.^56^ Studies in patients with AID have similarly suggested that imbalances in Treg number or function can contribute to AID, including rheumatoid arthritis, inflammatory bowel disease, and diabetes mellitus.^57^–^59^ For example, deletion of Treg in susceptible strains of mice accelerates the development of type 1 diabetes mellitus.^60^ The loss of Treg is associated with loss of suppression of T effector cells (Teff). Loss of Treg also leads to increased expression of adhesion molecules and chemokine receptors on Teff, leading to increased trafficking of Teff cells to the pancreas and increased destruction of beta cells.^61^

Similarly in multiple sclerosis (MS), loss of Treg leads to activation of autoreactive Teff cells and myelin destruction.^62^ Research in mouse models of experimental allergic encephalomyelitis (EAE), a model of human MS, has demonstrated that loss of Treg leads to development of EAE and that adoptive transfer of Foxp3^+^ Treg can ameliorate disease activity.^62^ Furthermore, therapies used to treat patients with MS, including glatiramir acetate and interferon (IFN)-β, lead to increases in Foxp3^+^ Treg and reduction in disease relapse.^63^,^64^ Accumulating data from patients with rheumatoid arthritis (RA) also suggest that dysregulation of Treg leads to development of RA.^65^ Finally, studies in experimental and human inflammatory bowel disease (IBD) shows that these diseases are Teff cell-driven and can be ameliorated by Treg.^58^

We have demonstrated that FGL2 has a role in autoimmune disease based on the finding that *fgl2*^−/−^ mice develop autoimmune glomerulonephritis. Importantly, Treg from *fgl2*^−/−^ mice have reduced suppressive activity.^13^ In humans, increased FGL2 plasma levels are associated with active autoimmune disease. These increased levels of FGL2 may be related to large numbers of Treg that are recruited to sites of inflammation in immunocompetent humans. Analysis of mucosal biopsies in patients with IBD has revealed that colitis flares are associated with elevated levels of FGL2.^66^ Higher levels of FGL2 are also found in synovial fluid from patients with rheumatoid arthritis compared with synovial fluid from patients with osteoarthritis.^67^

In order to study further the role of FGL2 in autoimmune disease, we utilized the T cell adoptive transfer model of IBD.^68^ In this model, *Rag1*^−/−^ mice develop a severe pan-colitis following transfer of CD4^+^CD25^−^CD45RB^hi^ T effector cells. The effect of CD4^+^CD25^+^CD45RB^lo^ Treg on colitis was then studied by co-administering these cells with the T effectors. We found that Treg from mice that ubiquitously overexpressed FGL2 (*fgl2*^Tg^ mice) completely prevented T cell-mediated colitis, whereas wild-type Treg were only partially protective, and Treg from *fgl2*^−/−^ mice were unable to prevent development of colitis (unpublished data). We also showed that T effector cells from *fgl2*^Tg^ mice were hypoproliferative and unable to induce colitis when injected into *Rag1*^−/−^ mice. These data support the concept that subsets of Treg expressing high levels of FGL2 are highly suppressive and critical for the development and maintenance of tolerance.

## ROLE OF TREG AND FGL2 IN VIRAL INFECTIONS

There is now mounting evidence that effective innate and adaptive immune responses are critical for viral clearance and the generation of long-lasting immunity to viral infections.^70^ The production of inhibitory factors can prevent the host from clearing viruses, resulting in chronic viral infection. Chronic hepatitis B virus (HBV), hepatitis C virus (HCV), and human immunodeficiency virus (HIV) infections are known to enhance the induction and proliferation of Treg.^70^–^72^ A number of investigators have reported increased numbers of Treg present in patients with chronic HBV and HCV infection when compared with successfully treated and/or healthy controls.^73^,^74^ Furthermore, *in vitro*, depletion of these cells increases virus-specific T cell responsiveness. Production of a variety of immunoregulatory cytokines such as TGF-β, IL-10, IL-35, and FGL2 has been proposed as an important mechanism by which Treg mediate their immunosuppressive activity.^12^,^21^

We and others have shown that FGL2 contributes to the pathogenesis of a number of experimental infectious diseases including mouse hepatitis virus strain 3 (MHV-3) infection and acute viral hepatitis caused by lymphocytic choriomeningitis virus (LCMV) WE strain.^75^,^76^ In an experimental model of fulminant hepatic failure caused by MHV-3, we showed that increased plasma levels of FGL2 as well as increased frequencies of FGL2-expressing Treg are predictive of susceptibility and severity of disease.^75^ Furthermore, inhibition of FGL2 by antibody or siRNA has been shown to protect susceptible animals fully from the lethality of MHV-3,^75^,^77^ whereas adoptive transfer of wild-type (WT) Treg into resistant *fgl2*^−/−^ animals accelerated their mortality.^77^ We have also examined the role of FGL2 in in a self-limiting murine model of acute viral hepatitis caused by LCMV WE. Following infection, plasma levels of FGL2 in wild-type C57BL/6 mice increased from a baseline of 0.8 ng/mL to a peak of 7.8 ng/mL at day 8 and remained elevated to day 50 post-infection.^76^ In order to characterize further the role of FGL2 in LCMV WE, we infected both wild-type and *fgl2*^−/−^ mice. Compared to wild-type mice, the *fgl2*^−/−^ mice displayed enhanced DC maturation, increased frequencies of virus-specific IFN-γ CD8^+^ T cells, and higher titers of virus-specific neutralizing antibody.^76^ These data demonstrate that FGL2 attenuates anti-viral responses and that therapeutic approaches to inhibit FGL2 may strengthen antiviral immune responses.

Studies from our laboratory and others have furthermore suggested that FGL2 is also involved in the pathogenesis of human chronic HBV and HCV infection.^78^,^79^ Patients with chronic HBV disease have been reported to have elevated plasma levels of FGL2 and increased expression of *fgl2* mRNA in their liver.^80^ Similarly, we recently reported that increased plasma levels of FGL2 in chronically infected HCV patients are associated with increased severity of liver disease and a poor outcome to anti-viral therapy.^78^ Current studies are being designed to evaluate the use of antibody to FGL2 and its receptor FcγRIIB in patients with chronic HBV and HIV infection.

## ROLE OF TREG AND FGL2 IN CANCER

Although Treg are known to regulate immune responses to cancer, the molecular mechanisms by which Treg are recruited to tumors and allow tumors to evade the immune system are not fully understood.^81^ Given its role as a Treg effector molecule, FGL2 has been shown to play a role in inhibiting anti-tumor immune responses. Microarray analyses have identified that *fgl2* is overexpressed in giant cell astrocytomas of the brain, as well as both low- and high-grade serous ovarian carcinomas.^82^ Some investigators have suggested that the prothrombinase activity of membrane-bound FGL2 is an important mediator of tumor angiogenesis and growth.^83^ However, two recent reports identified the immunoregulatory activity of soluble FGL2 as a key factor that inhibits the anti-tumor immune response. The first study examined the potential of a DC-based vaccine in a murine renal cell carcinoma model and demonstrated high *fgl2* expression in tumors that were not responsive to the vaccine.^84^ A second report investigated the role of FGL2 in glioblastoma multiforme (GBM), a highly aggressive type of brain cancer.^85^ Using human samples of GBM and low-grade gliomas, they demonstrated that GBM tumors have higher *fgl2* gene copy numbers than low-grade gliomas and that higher expression of the *fgl2* gene was associated with worse prognosis. A positive correlation was also demonstrated between *fgl2* expression in GBM and the expression of other immunomodulatory genes including *PD-1*, *PD-L2*, *CD39*, *BTLA*, *LAG-3*, *IL-10*, and *TGF-β1*.^85^ Using a GBM model in which delayed brain tumor (DBT) glioma cells are injected into mice, the investigators also demonstrated that CD4^+^CD39^+^Foxp3^+^ Treg were increased when FGL2 was overexpressed by the tumor cells. Interestingly, FGL2-expressing tumors also had increased numbers of immunomodulatory or alternatively activated M2 macrophages and myeloid-derived suppressor cells. Finally, in mice developing tumors injected with GL261 glioma cells overexpressing FGL2, treatment with anti-FGL2 monoclonal antibody resulted in prolonged survival versus mice treated with an isotype control antibody.^85^ These data suggest that FGL2 plays a role in modulating immune responses in cancer and that FGL2 may be a new target for cancer immunotherapy.

## TREG GENES AS BIOMARKERS FOR TOLERANCE

**Figure 4:**
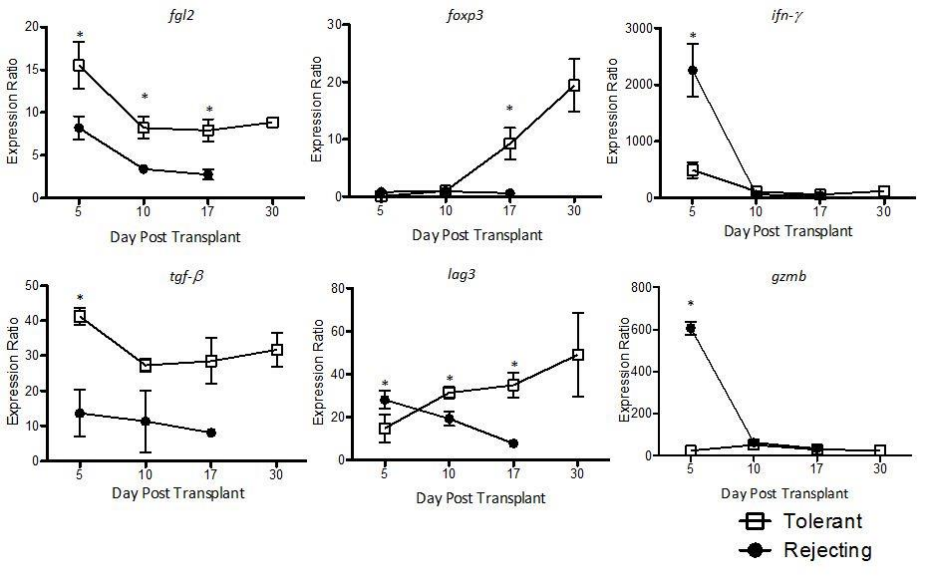
Differentially Expressed Treg-related Genes in Cardiac Allografts Serve as Putative Biomarkers of Tolerance Graphs display differentially expressed genes between tolerant (□) and rejecting (●) grafts from a panel of 22 Treg-related genes as assessed by multiplex RT-PCR. The expression of a gene was normalized to the housekeeping gene hypoxanthine phosphoribosyl transferase, and expression was then calculated as a ratio compared with the expression in non-transplanted hearts. Three allografts were used for each time point for both tolerant and rejecting groups. Graph shows mean±SEM. *P<0.05 versus rejecting group at the same time point. Copyright (2014) John Wiley and Sons. Used with permission from Urbanellis P, Shyu W, Khattar R, et al.^49^

Identification of transplant tolerance would be of great benefit to transplant recipients as these patients could be weaned off immunosuppression, sparing them from the side effects these medications.^86^ We have developed a gene expression biomarker panel that can distinguish between tolerance and rejection in animal models of transplantation.^49^ This panel includes 22 genes that have either anti-inflammatory (Treg-associated) or pro-inflammatory associations. As previously described, we succeeded in generating tolerance in mouse heart transplant model using a short course of rapamycin. We applied our biomarker panel to this model and found that intragraft expression of six genes can distinguish between tolerant and rejecting allografts (Figure 4). Tolerant allografts have high expression of the Treg-associated genes (*fgl2*, *foxp3*, *tgf*-β, and *lag-3*) and low expression of pro-inflammatory genes (*gzmb* and *ifn-*γ). In rejecting allografts, expression of these genes is reversed, with low expression of Treg-associated genes and high expression of pro-inflammatory genes. These data further support a role for Treg and FGL2 in allograft tolerance and demonstrate the utility of a gene biomarker panel that is based on these markers.

This same panel has also been studied in a mouse model of spontaneous liver allograft tolerance.^87^ In this liver transplant model, the allografts are initially characterized by histological evidence of severe cellular rejection, but over time the cellular rejection resolves and the grafts then appear histologically normal. At early time points (days 8–14), both Treg-associated and pro-inflammatory genes are upregulated in liver allografts. At a later time point, when the cellular rejection has resolved, expression of the pro-inflammatory genes was dramatically decreased but expression of the Treg genes, including *fgl2*, remained high. Interestingly, expression of these genes in the allograft but not in splenic mononuclear cells was predictive of tolerance, suggesting that local expression of these genes in the allograft may be critical for tolerance. We are now in the process of expanding this gene panel to the clinic to determine if it has utility as a biomarker for identifying tolerant liver transplant recipients. In the Liver Immune Tolerance Marker Utilization Study (LITMUS) that we are conducting, the profile will be utilized to identify transplant patients who may be tolerant. Proof of tolerance will be obtained by the ability to eliminate immunosuppression under careful monitoring using the biomarker gene expression profile described above.

Plasma levels of FGL2 may also have a role in identifying tolerant transplant recipients. In the mouse heart transplant model, we observed that tolerant mice had higher plasma levels of FGL2 at later time points (>30 days) than rejecting recipients, which may be related to upregulation of Treg in tolerant mice. Interestingly, the highest levels of FGL2 were observed in rejecting mice during acute rejection, which coincided with large influx of T cells into the graft.^49^ This increase in plasma FGL2 may represent a host response to control inflammation within the allograft. Thus, plasma levels of FGL2 may not be able to distinguish between tolerance and rejection at earlier time points but may be of utility in the long-term.

## HARNESSING THE CLINICAL POTENTIAL OF TREG AND THE FGL2–FCΓRIIB PATHWAY

Expansion of Treg has enormous clinical potential to treat conditions that are characterized by an underlying inflammatory process. Clinical trials are already underway to determine if Treg expanded *in vitro* and then transferred to patients can improve outcomes in solid organ transplantation and in patients with autoimmune disease. The ONE Study, for example, is a multicenter trial that is designed to compare the efficacy of CD4^+^CD25^+^ Treg and other types of cells with regulatory properties in renal transplantation.^88^ In autoimmunity, multiple trials are currently underway to assess Treg as therapeutic agents in rheumatoid arthritis, systemic lupus erythematosus, and IBD.^89^,^90^ In all of these trials there are important considerations which will need to be addressed, such as how the Treg are expanded *in vitro*, whether or not the Treg are antigen-specific, the stability of the Treg population, numbers of Treg to be transferred to the patient, and the timing of administration of Treg.^91^

Another consideration is that Treg are not homogeneous but are comprised of subsets with distinct suppressive properties. Based on the work of Joller et al. and work from our laboratory, TIGIT^+^FGL2^+^ Treg are a highly suppressive population of cells that can promote tolerance in autoimmunity and solid organ transplantation.^21^ We propose that isolation and transfer of this Treg subset would have additional clinical benefit beyond the transfer of a non-selected Treg population. Alternatively, Treg could be engineered *in vitro* to overexpress FGL2 prior to transfer to patients. These FGL2 high-expressing Treg would have enhanced suppressive properties compared with unmanipulated Treg. Given the obstacles in expanding Treg *in vitro*, it would be advantageous to generate FGL2-overexpressing Treg because fewer of these cells would be needed to achieve a clinical effect.

Although a short course of rFGL2 did not induce tolerance in our murine transplant models, rFGL2 may have an important therapeutic role in transplant medicine and in patients with autoimmune disease. As described above, we have generated purified rFGL2 that can prevent rejection of both skin and heart allografts in mice. In transplantation, rFGL2 may have a role as an induction agent in the immediate post-transplant setting. Our *in vivo* studies have revealed that rFGL2 treatment is not nephrotoxic and could therefore be used to prevent rejection and allow for renal recovery following solid organ transplantation. The rFGL2 would be preferable to other agents in that it is a recombinant product and would be expected to have less batch-to-batch variability than other induction agents such as thymoglobulin. Recombinant FGL2 may also have an important role in treating antibody-mediated rejection, where there is a need for new therapeutic agents that target antibody production. Recombinant FGL2 would be expected to have overlapping effects with intravenous immunoglobulin (IVIG) as they both have been shown to act through FcγRIIB, but rFGL2 would have the advantage of being a recombinant product and not generated from pooled human serum. Given the relatively large size of rFGL2 in its oligomeric form, we are now developing monomeric rFGL2 as a therapeutic agent. We have already demonstrated that monomeric rFGL2 is more efficacious than oligomeric rFGL2 in inhibiting immune responses *in vitro* and are now testing its immunomodulatory properties *in vivo*.^30^

Blocking the FGL2–FcγRIIB pathway with therapeutics may provide a novel treatment for chronic viral infections and cancer. The ability to evade immune monitoring and control is now considered an important functional hallmark of cancer.^92^,^93^ The CD4^+^CD25^+^Foxp3^+^ Treg are observed in several tumors and play a vital role in immune suppression of effector T cells.^94^ Hence, it can be posited that monoclonal antibodies against FGL2 may hinder Treg-mediated immune suppression in cancer. Moreover, current dendritic cell-based cancer vaccines are promising but fail to produce long-term anti-tumor immune response.^95^ Therefore, as previously demonstrated, the role of FGL2 in preventing DC maturation warrants the use of anti-FGL2 mAb as potential adjuvants for DC-based vaccines for various cancers. Finally, the role of FGL2 in many cancers has not yet been studied. Gene expression analyses of human tumor samples will be necessary to identify cancers where FGL2 is overexpressed and is inhibiting host anti-cancer immunity.

**Figure 5:**
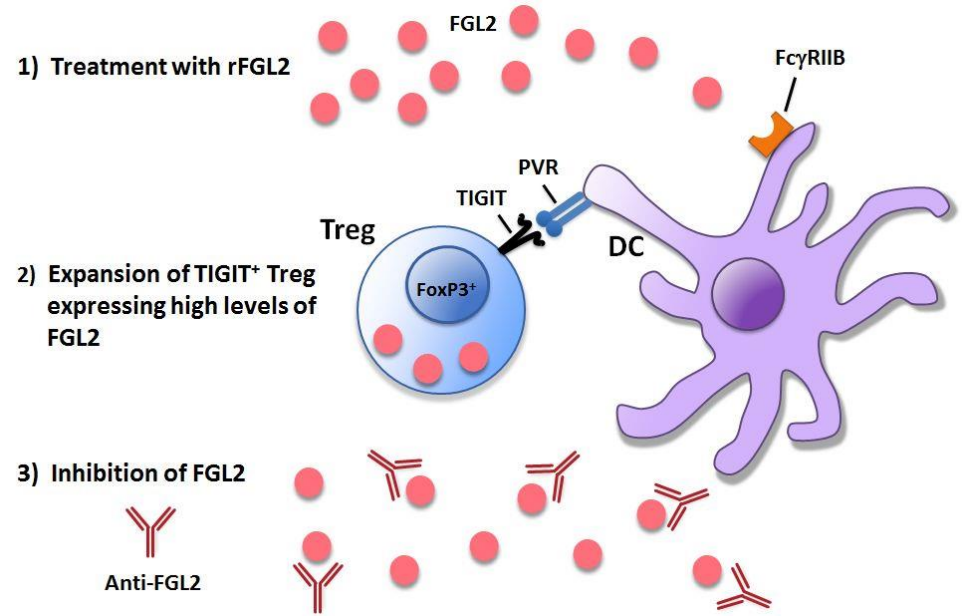
Potential Therapeutics Targeting the FGL2–FcγRIIB Pathway There are three main approaches currently being pursued to modulate the FGL2–FcγRIIB pathway for clinical benefit: (1) development of rFGL2 (monomeric and oligomeric) to inhibit immune responses; (2) expansion of TIGIT^+^ Treg expressing high levels of FGL2, which would represent a cellular therapy for transplantation and autoimmune disease; and (3) development of anti-FGL2 monoclonal antibodies to inhibit FGL2 signaling and enhance immune responses in cancer and chronic infections. DC, dendritic cell; PVR, poliovirus receptor; rFGL2, recombinant FGL2; TIGIT, T cell immunoreceptor with Ig and ITIM domains; Treg, regulatory T cell.

In addition to cancer therapy, therapeutics targeting FGL2 may also prove useful in treatment of infectious disease. Our previous studies demonstrated improved DC maturation and T cell responses to LCMV WE in *fgl2*^−/−^ mice versus wild-type infected mice.^76^ In addition to viral disease, targeted deletion of FGL2 also demonstrated improved outcomes in mice infected with *Echinococcus multilocularis*, a metacestode that causes alveolar echinococcis in humans and mice.^96^ Using mice provided by our laboratory, the authors were able to demonstrate reduced parasite loads and increased T cell responses in *fgl2*^−/−^ mice. Thus, anti-FGL2 mAb may prove to be useful in treatment of chronic viral disease as well as parasitic infections. This therapy may hold the potential for using the host immune response against parasites and viruses as opposed to current antiviral and anti-helminthic drugs. Furthermore, patients with impaired immune systems may benefit from anti-FGL2 antibody therapy as an adjuvant to improve immune responses to vaccines. Potential therapies based on modulating the FGL2–FcγRIIB pathway are highlighted in Figure 5.

In conclusion, the FGL2–FcγRIIB pathway is a critical immunoregulatory pathway that is involved in alloimmunity, autoimmunity, chronic infections, and cancer. Therapies based on either augmenting or inhibiting this pathway hold great promise in treating these diverse medical conditions.

## Abbreviations

AID: autoimmune disease
APC: antigen-presenting cells
BMDC: bone marrow-derived dendritic cell
DC: dendritic cells
EAE: experimental allergic encephalomyelitis
FcγR: Fcγ receptor
FGL2: fibrinogen-like protein 2
FRED: fibrinogen-related domain
GBM: glioblastoma multiforme
HBV: hepatitis B virus
HCV: hepatitis C virus
HIV: human immunodeficiency virus
IBD: inflammatory bowel disease
IL: interleukin
IVIG: intravenous immunoglobulin
LCMV: lymphocytic choriomeningitis virus
MHV: mouse hepatitis virus
MLR: mixed lymphocyte reaction
MS: multiple sclerosis
PBMC: peripheral blood mononuclear cells
TIGIT: T cell immunoreceptor with Ig and ITIM domains
Teff: effector T cells
Treg: regulatory T cells.
